# Neutralizing Antibodies Against Factor VIII Can Occur Through a Non-Germinal Center Pathway

**DOI:** 10.3389/fimmu.2022.880829

**Published:** 2022-05-11

**Authors:** Seema R. Patel, Taran S. Lundgren, Wallace Hunter Baldwin, Courtney Cox, Ernest T. Parker, John F. Healey, Ryan P. Jajosky, Patricia E. Zerra, Cassandra D. Josephson, Christopher B. Doering, Sean R. Stowell, Shannon L. Meeks

**Affiliations:** ^1^ Aflac Cancer and Blood Disorders Center, Children’s Healthcare of Atlanta/Emory University School of Medicine, Atlanta, GA, United States; ^2^ Graduate Program in Molecular and Systems Pharmacology, Laney Graduate School, Emory University School of Medicine, Atlanta, GA, United States; ^3^ Brigham and Women’s Hospital, Harvard Medical School, Boston, MA, United States; ^4^ Center for Transfusion Medicine and Cellular Therapies, Department of Laboratory Medicine and Pathology, Emory University School of Medicine, Atlanta, GA, United States

**Keywords:** hemophilia A, germinal center, B cells, neutralizing antibodies (inhibitors), extrafollicular pathway

## Abstract

Humoral immunity to factor VIII (FVIII) represents a significant challenge for the treatment of patients with hemophilia A. Current paradigms indicate that neutralizing antibodies against FVIII (inhibitors) occur through a classical CD4 T cell, germinal center (GC) dependent process. However, clinical observations suggest that the nature of the immune response to FVIII may differ between patients. While some patients produce persistent low or high inhibitor titers, others generate a transient response. Moreover, FVIII reactive memory B cells are only detectable in some patients with sustained inhibitor titers. The determinants regulating the type of immune response a patient develops, let alone how the immune response differs in these patients remains incompletely understood. One hypothesis is that polymorphisms within immunoregulatory genes alter the underlying immune response to FVIII, and thereby the inhibitor response. Consistent with this, studies report that inhibitor titers to FVIII differ in animals with the same *F8* pathogenic variant but completely distinct backgrounds; though, how these genetic disparities affect the immune response to FVIII remains to be investigated. Given this, we sought to mechanistically dissect how genetics impact the underlying immune response to FVIII. In particular, as the risk of producing inhibitors is weakly associated with differences in HLA, we hypothesized that genetic factors other than HLA influence the immune response to FVIII and downstream inhibitor formation. Our data demonstrate that FVIII deficient mice encoding the same MHC and *F8* variant produce disparate inhibitor titers, and that the type of inhibitor response formed associates with the ability to generate GCs. Interestingly, the formation of antibodies through a GC or non-GC pathway does not appear to be due to differences in CD4 T cell immunity, as the CD4 T cell response to an immunodominant epitope in FVIII was similar in these mice. These results indicate that genetics can impact the process by which inhibitors develop and may in part explain the apparent propensity of patients to form distinct inhibitor responses. Moreover, these data highlight an underappreciated immunological pathway of humoral immunity to FVIII and lay the groundwork for identification of biomarkers for the development of approaches to tolerize against FVIII.

## Introduction

Hemophilia A is an X-linked bleeding disorder resulting from a deficiency in the blood coagulation protein factor VIII (FVIII). As prophylaxis, most patients with severe hemophilia A receive either regular infusions of FVIII or emicizumab, a FVIII mimetic. Moreover, to treat bleeding episodes or provide protection for surgeries, patients are administered additional doses of FVIII in combination with either prophylaxis regimen. However, recurrent exposure to FVIII can lead to the development of inhibitors (neutralizing antibodies to FVIII), with 20-30% of patients with severe and 5% of patients with mild to moderate hemophilia A producing inhibitors within the first 50 exposure days to FVIII (1, 2). Inhibitors mitigate the therapeutic benefits of FVIII by impeding its procoagulant activity and are a crippling barrier for gene therapy. As a result, inhibitors increase morbidity and mortality, increase cost of care, and decrease quality of life for this patient population (3–6). Unfortunately, no strategies currently exist to prevent the formation of inhibitors or mediate tolerance to FVIII prior to the development of immunity in previously untreated patients with hemophilia A. This in part stems from a fundamental lack of understanding regarding key factors that regulate the B cell response to FVIII. In addition, it is not completely understood why a large population of patients never form inhibitors and demonstrate tolerance to FVIII. Understanding the mechanisms by which inhibitors develop may aid in the identification of critical targets that can be exploited to prevent inhibitors or induce tolerance to FVIII in patients with hemophilia A.

Several clinical and preclinical studies suggest that inhibitors develop through a classical CD4 T cell dependent process (7–11), wherein T follicular helper cells (TFH) work in concert with cognate follicular B cells to drive a germinal center (GC) reaction that is ultimately responsible for the propagation and selection of affinity matured, class-switched (CSW) memory B cells and long-lived plasma cells. However, clinical observations like those reported in the recent HIPS (Hemophilia Inhibitor Previously Untreated Patient) study (12) suggest that the nature of the immune response to FVIII differs between patients. While some patients form sustained low (<5 Bethesda Units) or high (>5 Bethesda Units) inhibitor titers, others produce transient low titers that resolve within 6 months and without any therapeutic interventions (12–14). Moreover, there are a group of individuals that primarily develop non-neutralizing IgG1 with low-affinity for FVIII (12, 13); these patients never develop antibodies with inhibitory activity. In addition, clinical studies evaluating the immune response to FVIII demonstrate that FVIII specific memory B cells are not detectable in all patients with a history of persistent inhibitors (15–17), though how this compares to patients with transient inhibitors has yet to be explored. Furthermore, patients with existing inhibitors respond differently to immune tolerance induction (ITI) therapy, a treatment that entails higher and often more frequent doses of FVIII: (1) “tolerance” - undetectable inhibitor titers following return to “normal” FVIII pharmacokinetic doses, (2) “partial tolerance” - absence of anamnesis when exposed to “normal” FVIII doses or (3) “failure” - persistent inhibitor titers that prevent use of FVIII therapy (18–20). Interestingly, some patients indefinitely remain “tolerant” or “partially tolerant”, while others relapse following withdrawal of ITI therapy or when exposed to “normal” FVIII pharmacokinetic doses in the presence of inflammation or during puberty (3, 21).

The primary determinants regulating the type of immune response a patient will develop to FVIII, let alone how the immune response mechanistically differs in these patients remains incompletely understood. One hypothesis is that genetic factors associated with an increased risk of forming inhibitors may likewise contribute to the development of distinct immune responses to FVIII (22–30). For instance, the type of *F8* variant a patient encodes has been found to correlate with an increased propensity to develop inhibitors (22, 23); there are over 2,000 *F8* pathogenic variants that can cause severe, mild or moderate hemophilia A depending on the degree to which the variant impacts formation of active FVIII. While 60-70% of hemophilia A patients with large deletions/insertions (>50 base pairs and +1 exons) in *F8* generate inhibitors, roughly 15% of individuals with small deletions/insertions (<50 base pairs) produce inhibitors (23). The relative risk of a patient forming inhibitors according to the type of *F8* variant the individual encodes is proposed to be determined by whether the pathogenic variant allows for production of partial or inactive FVIII, also referred to as cross-reactive material (CRM). This CRM may generate some degree of central tolerance that can not only protect against the initiation of a humoral immune response to FVIII but also regulate immunity once onset (22, 23). Moreover, immune polymorphisms within genes encoding immunoregulatory molecules (e.g. *IL10, TNFA and Ctla4*) and Human Leukocyte Antigen (HLA) variants possess the ability to modulate the immune response to FVIII by altering immune activation thresholds and/or differentiation of regulatory cells (24–30). Consistent with this, preclinical studies report that inhibitor titers to recombinant and transgene FVIII differ in FVIII deficient mice or canines on completely disparate genetic backgrounds (31–33). However, how these genetic disparities affect the immune response to FVIII and downstream inhibitor outcome remains to be evaluated. Given this, we sought to determine how genetics influence the mechanistic underpinnings of the immune response to FVIII. As siblings with the same *F8* pathogenic variant can have discrepant inhibitor responses and the risk of forming inhibitors is weakly associated with HLA variants (28, 29, 34), we hypothesized that genetic factors other than HLA and *F8* affect the underlying immune response to FVIII, and thereby contribute to the distinct inhibitor signatures observed in patients with hemophilia A.

As mechanistic studies testing the role of genes in immunity to FVIII in patients with hemophilia A are not feasible, preclinical models of hemophilia A that encode the same *F8* pathogenic variant and Major Histocompatibility Complex (MHC) haplotype but are on disparate genetic backgrounds [C57BL/6J (B6) versus mixed S129/B6 (129S4/SvJae + B6)] (35, 36) were employed. Our data demonstrate that B6 and S129/B6 FVIII deficient mice form different IgG and inhibitor titers to FVIII, and that the B cell response to FVIII in these mice may occur through two distinct immune pathways. These results thus suggest that genetic factors apart from MHC and *F8* may regulate the process by which humoral immunity to FVIII occurs. Moreover, these data for the first time provide mechanistic insight into how genetics influence the immune response to FVIII as well as highlight a novel pathway by which inhibitors can form to FVIII. In addition, these findings establish a foundation for identification of biomarkers for the generation of therapeutic approaches to promote immune tolerance to FVIII in all patients with hemophilia A.

## Methods

### Mice

FVIII deficient mice with a disruption in the exon 16 of the *F8* gene were obtained as F1 hybrids [S129 + B6] from L. W. Hoyer (Holland Laboratory) (35). Mice were either backcrossed 5 (S129/B6; mixed 70% B6 and 30% S129 background) or >10 generations onto a B6 background (>97% B6 background) (36). Eight to 12-week old male and female mice were used. Mice were housed and bred in the Emory University Division of Animal Resources facilities. All procedures were performed according to approved Institutional Animal Care and Use Committee protocols.

### Antibodies

Alkaline Phosphatase (AP) anti-mouse IgM, IgG1, IgG2a, IgG2b, IgG2c and IgG3 were acquired from Southern Biotech, while AP anti-mouse IgG was purchased from Bio-rad. BV421 anti-mouse I-A^b^, PE anti-mouse I-A^d^, BV421 anti-mouse H-2K^b^, BV421 anti-mouse GL7, BV480 anti-mouse IgM, BV510 anti-mouse CD38, BB515 anti-mouse CD19, BV650 anti-mouse RORγt, PE CF594 anti-mouse GATA3, BB515 anti-mouse CD8α, and Alexa Fluor 647 (AF647) anti-mouse Bcl6 were obtained from BD Biosciences. FITC anti-mouse I-A^k^, AF700 anti-mouse CD45, BV510 anti-mouse H-2K^d^, PE anti-mouse H-2K^k^, BV605 anti-mouse CD19, AF700 anti-mouse T cell receptor (TCR), AF700 anti-mouse CD11b, AF700 anti-mouse CD11c, BV650 anti-mouse IgD, BV421 anti-mouse CD3, BV510 anti-mouse CD4, BV605 anti-mouse Tbet, BV785 anti-mouse CD25, PerCP Cy5.5 anti-mouse CD19, PerCP Cy5.5 anti-mouse CD11c, PerCP Cy5.5 anti-mouse CD11b, and AF700 anti-mouse CD44 were purchased from Biolegend.

### Recombinant B Domain Deleted FVIII

B domain deleted human FVIII was prepared as previously described (37). Briefly, the B domain of FVIII was replaced with an “SQ” linker (SFFQNPPVLKRHQR) using site specific mutagenesis. The “RHQR” amino acid sequence of the SQ linker is a recognition site for PACE/furin, and thereby facilitates intracellular processing of FVIII from a single chain to a heterodimer (37, 38). The modified FVIII cDNA was then cloned into a mammalian expression vector (pIRES/Puro) and transfected into baby hamster kidney cells. Clones expressing the highest level of FVIII were selected and expanded, followed by purification of FVIII from cell culture supernatant. FVIII concentration was calculated using an extinction coefficient at A_280_ of 1.53 mg/mL^-1^ cm^-1^ (39).

### Analysis of Antibody and Inhibitor Response to FVIII

S129/B6 and B6 FVIII deficient mice were administered 4 weekly retro-orbital infusions of 1 μg FVIII. Two weeks after the last infusion, mice were challenged with 2 μg FVIII. Plasma was collected 7 days post each injection for measurement of total anti-FVIII IgM, IgG or IgG subclasses (IgG1, IgG2a, IgG2b, IgG2c, IgG3) by an enzyme-linked immunosorbent assay (ELISA) (36, 40). The ELISA was performed using microtiter plates coated with FVIII and plasma was serially diluted 3.5-fold starting at a 1:20 dilution. AP anti-mouse IgM, IgG, or IgG subclasses diluted 1:1000 were then used to detect bound antibodies. ELISA titration curves were fitted to the 4-paramter logistic equation and a positive titer was defined as a dilution that produced an A_405_ of 0.3. Inhibitor titers were measured by a modified Nijmegen Bethesda assay (40–43). The Bethesda assays were performed by reconstituting human FVIII deficient plasma with the same FVIII that was infused into mice. One Bethesda Unit (BU) per mL was defined as the dilution of plasma that results in 50% inhibition of FVIII activity. An inhibition curve was fitted using the 4-paramter logistic equation to estimate the concentration of IgG producing 50% inhibition.

### FVIII and Hen Egg Lysozyme (HEL) B Cell Tetramer Production

To create a FVIII B cell tetramer, B domain deleted human FVIII was modified using site-directed mutagenesis to include a cysteine substitution (K1804C) in the A3 domain (44); the amino acid numbering of the cysteine is based on the sequence of full-length FVIII. The FVIII variant was reduced with TCEP (Thermofisher Scientific) for 30 minutes on ice to uncap the cysteine residue, and then allowed to recover overnight at 4°C. An EZ-link Maleimide-PEG2-Biotin kit (Thermofisher Scientific) was used to biotinylate the uncapped cysteine residue. Free biotin was removed by centrifugation in a Zeba spin desalting column (Thermofisher Scientific) and the molar ratio of biotin to FVIII was quantified using a Biotin Quantitation kit (Thermofisher Scientific). Streptavidin conjugated to phycoerythrin (SA-PE; Biolegend) was then added to the biotinylated FVIII at a 1 to 6 molar ratio for tetramerization. A HEL B cell tetramer was developed as previously described (45). Briefly, purified HEL (Worthington Biochemical) was biotinylated using an EZ-link Sulfo-NHS-LC-Biotinylation kit (Thermofisher Scientific). Biotinylated HEL with ≤1 biotin per molecule was then tetramerized using SA-PE. A “non-specific tetramer” was also created by labeling SA-PE with Alexa Flour 647 (AF647; Invitrogen) for 60 minutes at room temperature. Free AF647 was removed by centrifugation in a 100-kD cut off Amicon Ultra Filter (Millipore Sigma). Prior to use, the SA-PE*AF647 was incubated with 6-fold molar excess of 1 μM D-biotin. As the ratio of SA to PE is roughly 1:1, the concentration of each tetramer was calculated using an extinction coefficient at A_565_ of 1.96 μM^-1^ cm^-1^.

### Precursor Frequency of FVIII Reactive B Cells

The spleen and peripheral lymph nodes (inguinal, axillary, brachial, submandibular, and mesenteric) were harvested in IMDM media from naïve B6 and S129/B6 FVIII deficient mice. Cells were centrifuged at 1500 rpm for 5 minutes, and subsequently resuspended in IMDM media containing 2.4G2 Fc block (BD Bioscience) + 5 nM SA-PE*AF647 (1 μM D-biotin + SA-PE*AF647). Samples were incubated for 10 minutes at room temperature, followed by addition of 4 nM FVIII B cell tetramer and incubation on ice for 30 minutes. Cells were then washed with cold IMDM media and resuspended in IMDM media containing 50 μl anti-PE microbeads (Miltenyi Biotech). Samples were incubated on ice for 30 minutes and subsequently washed with cold IMDM media. Tetramer^+^ cells were then enriched by resuspending samples in 3 mL cold FACS buffer (1x DPBS + 2% bovine serum albumin) and passing the cells over a magnetized LS column (Miltenyi Biotech). Columns were washed two times with 3 mL cold FACS buffer and then removed from the magnet. A plunger was used to push 5 mL FACS buffer through the column for elution of bound tetramer^+^ cells. Following centrifugation, bound and unbound fractions were resuspended to 100 μl and 2 mL cold FACS buffer, respectively. To determine absolute counts, 5 μl from each sample were added to 200 μl AccuCheck counting beads (Invitrogen). The remaining cell suspensions were incubated for 30 minutes on ice with BV605 anti-mouse CD19 + AF700 anti-mouse TCR + AF700 anti-mouse CD11b + AF700 anti-mouse CD11c + Live/Dead Fixable Near-IR (Thermofisher Scientific). All samples were run on a 4 laser Cytek Aurora and analyzed using FlowJo software.

### Transfusion of HEL Sheep Red Blood Cells (HEL SRBCs)

To chemically link HEL to SRBCs, HEL was first activated with Sulfo-NHS (Thermofisher Scientific) and 1-ethyl-3-(3-dimethylaminopropyl)carbodiimide hydrochloride (EDC) in MES buffer for 15 minutes at room temperature. Activated HEL was passed over a Zeba spin desalting column equilibrated with PBS at pH 7.4, lyophilized and stored at -20°C until use. SRBCs (Rockland Laboratories) were washed in 1x DPBS and resuspended in 1x DPBS at a 2 to 1 ratio. Activated HEL was quickly thawed at room temperature and reconstituted in deionized water. Reconstituted activated HEL was immediately added at an equal volume to the packed SRBCs and incubated for 25 minutes at room temperature. The cells were washed in 1x DPBS. To confirm HEL linkage to SRBCs, HEL SRBCs and unlabeled SRBCs were stained with or without anti-HEL antibodies (clone: 2F4 and 4B7; Bioxcell) for 15 minutes at room temperature. Cells were washed and then resuspended in APC anti-mouse IgG for 15 minutes at room temperature. Samples were run on a 4 laser Cytek Aurora and analyzed using FlowJo software. B6 and S129/B6 FVIII deficient mice were transfused *via* the retro-orbital plexus with 50 μl packed HEL SRBCs diluted to a 150 μl total volume in 1x DPBS.

### Immunophenotyping the Immune Response to FVIII and HEL

As the spleen appears to be the primary site of inhibitor formation (46), splenocytes were harvested from B6 and S129/B6 FVIII deficient mice 7 days post transfusion of HEL SRBCs or challenge with 2 μg FVIII. To evaluate the B cell response, pelleted cells were stained with 2.4G2 Fc block + 5 nM SA-PE*AF647, followed by 4 nM FVIII B cell tetramer or 5 nM HEL B cell tetramer. To examine the CD4 T cell response, pelleted cells were resuspended in 2.4G2 Fc block + 18 μg/mL FVIII MHC Class II tetramer (I-A^b^ : FVIII_2210-2229_) and incubated for 1 hour at room temperature; the FVIII MHC Class II tetramer was provided by the National Institute of Allergy and Infectious Diseases Tetramer Core Facility at Emory University and using the TASSYFTNMFATWSPSKARL (47) FVIII peptide presented on the MHC Class II haplotype H-2^b^. All samples were then washed and anti-PE microbeads were added. Tetramer^+^ cells were enriched using the magnetized LS columns as described above. Bound fractions were resuspended to 100 μl cold FACS buffer and 5 μl was added to AccuCheck counting beads. For B cell immunophenotyping, remaining cell suspensions were incubated for 30 minutes on ice with BV421 anti-mouse GL7 + BV480 anti-mouse IgM + BV510 anti-mouse CD38 + BV650 anti-mouse IgD + BB515 anti-mouse CD19 + AF700 anti-mouse TCR+ AF700 anti-mouse CD11b+ AF700 anti-mouse CD11c+ Live/Dead Fixable Near-IR. For CD4 T cell immunophenotyping, remaining cells were stained for 30 minutes on ice with BV421 anti-mouse CD3+ BV510 anti-mouse CD4+ BV785 anti-mouse CD25+ BB515 anti-mouse CD8α+ PerCP Cy5.5 anti-mouse CD19+ PerCP Cy5.5 anti-mouse CD11c+ PerCP Cy5.5 anti-mouse CD11b+ AF700 anti-mouse CD44+ Live/Dead Fixable Near-IR. Polarization of the CD4 T cell response was investigated by fixing and permeabilizing cells post surface stain for 30 minutes on ice using eBioscience Foxp3/Transcription Factor Fixation/Permeabilization buffer (Thermofisher Scientific). Fixed and permeabilized cells were washed in 1x Permeabilization Wash Buffer (Thermofisher Scientific), and subsequently stained for 45 minutes at room temperature with AF647 anti-mouse Bcl6 + PE CF594 anti-mouse GATA3 + BV605 anti-mouse Tbet + BV650 anti-mouse RORγt. All samples were run on a 4 laser Cytek Aurora and analyzed using FlowJo software; mean fluorescent intensity (MFI) was used to assess expression of transcription factors.

### Statistics

Statistical analysis was performed using a non-parametric Mann-Whitney test or Kruskal-Wallis with a Dunn’s multiple comparison post-test. Significance was determined by a P value < 0.05.

## Results

### FVIII Deficient Mice With an Identical F8 Pathogenic Variant and MHC Haplotype but Different Genetic Backgrounds Develop a Distinct Antibody and Inhibitor Response to FVIII

**Figure 1:**
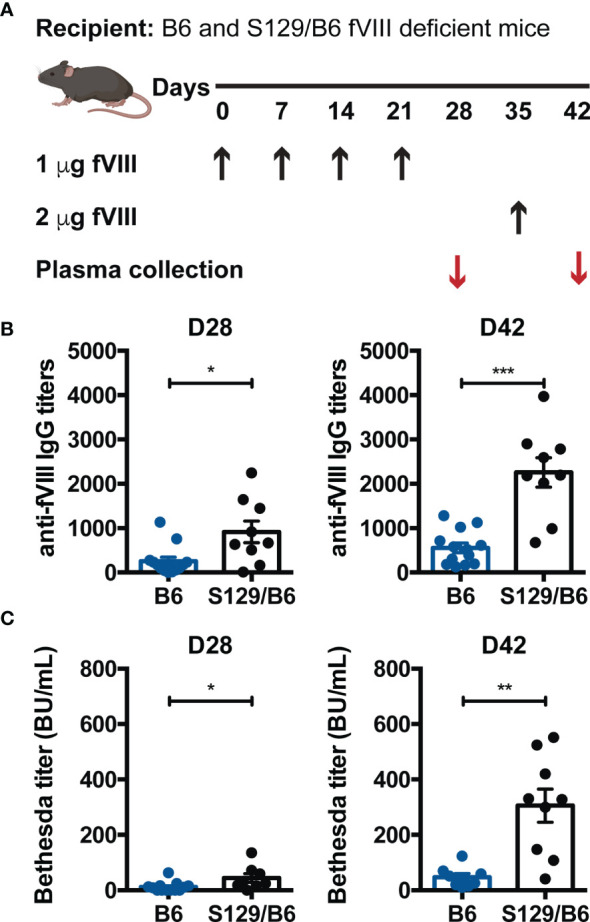
FVIII deficient mice on a B6 background generate lower anti-FVIII IgG and inhibitor titers than those on an S129/B6 background. **(A)** Experimental schematic of FVIII immunization. B6 and S129/B6 FVIII deficient mice were administered 4 weekly infusions of 1 μg FVIII, followed by a 2 μg challenge. Plasma was collected pre (day 28; D28) and post (day 42; D42) challenge, and evaluated for production of anti-FVIII IgG **(B)** as well as inhibitors **(C)** by ELISA and Bethesda assay, respectively. Error bars represent ± SEM. Statistics were generated using an unpaired Mann-Whitney test. There were 8-13 mice per group. Data shown are the combined results from 2 experiments. *p < 0.05, **p < 0.01, and ***p < 0.001.

To formally test the hypothesis that genetic factors other than HLA and *F8* impact the inhibitor response to FVIII, the relative immunogenicity of FVIII was examined in B6 and S129/B6 FVIII deficient mice that encode the same *F8* pathogenic variant and MHC haplotype (H-2^b^; **Supplementary Figure 1**) but are on distinct genetic backgrounds. This was accomplished by administering B6 and S129/B6 FVIII deficient mice 1 μg FVIII weekly for 4 weeks, followed by a 2 μg challenge (**Figure 1A**) (48). Mice were treated with 5 doses of FVIII, as the formation of antibodies and inhibitors to FVIII in both patients and preclinical models of hemophilia A are known to occur following multiple exposures to FVIII (12, 49, 50). Plasma was then collected one week post each infusion and measured for IgG reactive to FVIII by ELISA and inhibitors by a modified Bethesda Nijmegen assay.

Although 100% of B6 and S129/B6 FVIII deficient mice produced IgG specific to FVIII one week following challenge, B6 and S129/B6 FVIII deficient mice demonstrated disparate total anti-FVIII IgG titers pre and post challenge. S129/B6 FVIII deficient mice developed higher total IgG titers to FVIII compared to B6 FVIII deficient mice (**Figure 1B**). As studies indicate that the antibody response to FVIII consist of both inhibitors and non-neutralizing anti-FVIII IgG (12, 51), the total IgG response to FVIII may not reflect the titers of FVIII reactive antibodies with inhibitory activity. To determine whether B6 and S129/B6 FVIII deficient mice generate a differential inhibitor response to FVIII, plasma from treated mice was also tested for inhibitors. Consistent with the total IgG response to FVIII, 100% of both strains of FVIII deficient mice formed inhibitors one-week post challenge. Moreover, B6 FVIII deficient mice generated lower Bethesda titers pre and post challenge compared to S129/B6 FVIII deficient mice (**Figure 1C**). Combined, these data indicate that genetic differences stemming from non-MHC and -*F8* genes may play a role in regulating the humoral immune response to FVIII.

### Precursor Frequency of FVIII Reactive B Cells Is Slightly Lower in S129/B6 FVIII Deficient Mice

**Figure 2:**
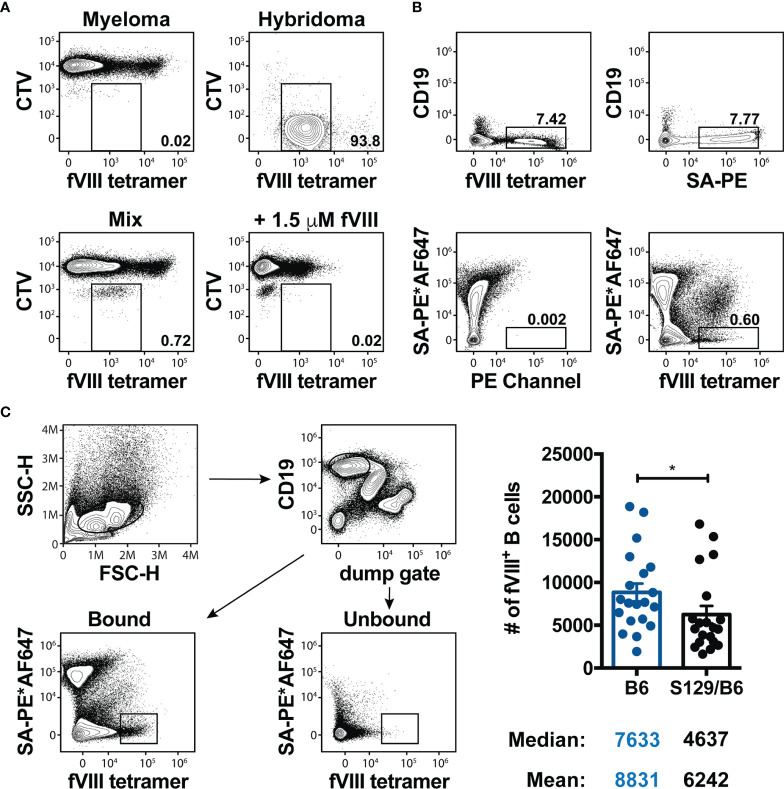
Precursor frequency of FVIII reactive B cells is slightly lower in S129/B6 FVIII deficient mice. **(A)** Representative contour plots illustrating the specificity of the FVIII B cell tetramer. 20,000 Cell Trace Violet (CTV) labeled hybridomas expressing a BCR reactive to FVIII were mixed with 2 x 10^6^ myeloma cells (fusion partner) that express an invariant BCR. Samples were then incubated with IMDM media or 1.5 μM full-length FVIII 10 minutes prior to the FVIII B cell tetramer. Flow cytometry was utilized to detect CTV^+^ hybridomas expressing a BCR specific to FVIII. **(B)** Representative flow cytometric analysis of B cells in a fraction enriched using anti-PE magnetic microbeads after staining with the FVIII B cell tetramer, SA-PE and/or the SA-PE*AF647 non-specific tetramer. **(C)** Gating strategy and graphical demonstration of the precursor frequency of FVIII specific B cells. Splenocytes and peripheral lymph nodes were harvested from naïve B6 and S129/B6 FVIII deficient mice, and subsequently enriched and quantified using the FVIII B cell tetramer. Error bars represent ± SEM. Statistics were generated using an unpaired Mann-Whitney test. There were 20 mice per group in panel **(C)**. Results illustrated are representative of 2-3 experiments **(A, B)** or combined data from 20 experiments **(C)**. *p < 0.05.

As pre-clinical studies demonstrate that the pre-immune B cell repertoire of wild type mice on disparate genetic backgrounds can be comprised of a different number of B cells reactive to the same immunogen (52) and the precursor frequency of B cells can impact the B cell response to an antigen (53), it is conceivable that the tendency of S129/B6 FVIII deficient mice to form an enhanced IgG and inhibitor response to FVIII is simply due to these mice having an initially higher number of FVIII specific B cells. As no immunological tools currently exist to detect naïve FVIII specific B cells, a FVIII B cell tetramer that permits flow cytometric identification of FVIII reactive B cells was engineered. This was accomplished by generating a FVIII variant with a single site mutation in the A3 domain (K1804C) of FVIII that permits mono-biotinylation of the FVIII variant (44); mono-biotinylation of the FVIII variant did not mask all currently identified epitopes within FVIII, thereby allowing for identification of the FVIII reactive B cell repertoire. The mono-biotinylated FVIII was then conjugated to a SA-PE to increase the avidity of the tetramer for FVIII specific B cell receptors (BCRs). As the FVIII B cell tetramer did not bind to Cell Trace Violet (CTV) labeled myeloma cells expressing an invariant BCR (54) and incubation with a large molar excess of monomeric FVIII prior to addition of the tetramer resulted in the loss of detection of tetramer^+^ hybridomas expressing a BCR specific to FVIII (**Figure 2A**), these results demonstrate that binding of the FVIII B cell tetramer is BCR specific. Moreover, these data indicate that the FVIII B cell tetramer serves as a novel tool to detect FVIII reactive B cells in a polyclonal repertoire.

Considering the precursor frequency of B cells of any given specificity is relatively rare and too low to be detected through conventional staining of a small sample of lymphocytes from the spleen and peripheral lymph nodes, antigen specific B cells from the entire spleen and peripheral lymph nodes are typically assessed by enriching for tetramer^+^ B cells using magnetic microbeads (52, 55–57). Accordingly, the precursor frequency of FVIII specific B cells in the pre-immune repertoire of B6 and S129/B6 FVIII deficient mice was quantified using this methodology. As the FVIII B cell tetramer contains PE, FVIII reactive B cells from the whole spleen and peripheral lymph nodes were enriched using anti-PE magnetic microbeads. Samples were then passed over a magnetized column and washed multiple times. The columns were removed and a plunger was used to flush out bound cells. B cells in the bound and unbound fractions were identified as cells lacking non-B cell lineage markers (CD11b, CD11c and TCR) but expressing the B cell marker CD19 (**Figure 2B**).

Enrichment of FVIII specific B cells demonstrated a sizeable population of FVIII tetramer^+^ B cells in the spleen of naïve FVIII deficient mice (**Figure 2B**). However, as mice also produce PE reactive B cells and the tetramer possesses the ability to bind B cells specific to SA or biotin within the FVIII B cell tetramer (55), it was unclear whether these FVIII tetramer^+^ cells were B cells specific to FVIII, PE, SA and/or biotin. Consistent with this, bound fractions from the spleen of naïve FVIII deficient mice stained with only SA-PE resulted in a percent frequency of B cells that were indistinguishable from those enriched using the FVIII B cell tetramer (**Figure 2B**). Thus, to identify FVIII specific B cells, samples were first incubated with a “non-specific tetramer” that was generated by labeling SA-PE with an irrelevant fluorophore (AF647) (56, 58), followed by addition of the FVIII B cell tetramer. As labeling SA-PE with AF647 generates a unique spectral footprint compared to PE alone, this approach allows for separation of FVIII reactive B cells from B cells that are specific to other components of the FVIII B cell tetramer (**Figure 2B**). Surprisingly, S129/B6 FVIII deficient mice that developed enhanced total anti-FVIII IgG and inhibitor titers demonstrated a slightly lower precursor frequency (median: 4637) of FVIII reactive B cells compared to B6 FVIII deficient mice (median: 7633) that had formed lower titers of total IgG and inhibitors to FVIII (**Figure 2C**). These results suggest that the precursor frequency of FVIII specific B cells may not contribute to the discordant inhibitor response observed in B6 and S129/B6 FVIII deficient mice.

### S129/B6 FVIII Deficient Mice Develop a More Robust GC B Cell Response to FVIII Than FVIII Deficient Mice on a B6 Background

**Figure 3:**
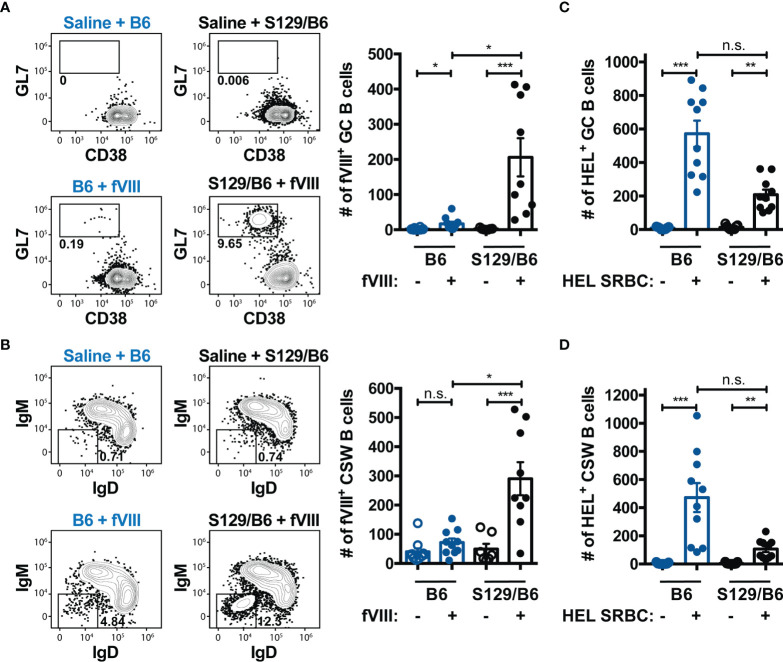
FVIII deficient mice on an S129/B6 background generate a robust GC and CSW B cell response to FVIII compared to B6 FVIII deficient mice. **(A, B)** Gating strategy and graphical illustration of the absolute counts of FVIII specific GC **(A)** and CSW **(B)** B cells in B6 and S129/B6 FVIII deficient mice administered saline or 4 weekly infusions of 1 μg FVIII, followed by a 2 μg challenge. Splenocytes were harvested and FVIII specific B cells were enriched and quantified using the FVIII B cell tetramer. **(C, D)** Number of HEL reactive GC **(C)** and CSW **(D)** B cells 7 days post transfusion of HEL SRBCs in B6 and S129/B6 FVIII deficient mice. HEL reactive B cells were enriched and enumerated using the HEL B cell tetramer. Error bars represent ± SEM. Statistics were generated using a Kruskal-Wallis with a Dunn’s multiple comparison post-test. There were 7-10 mice per group. The data demonstrated are the combined results from 2 experiments. *p < 0.05, **p < 0.01, ***p < 0.001, and n.s. indicates not significant.

Current paradigms indicate that humoral immunity to proteins or glycoproteins like FVIII occurs through a GC reaction, a specialized microanatomical structure within secondary lymphoid organs that are ultimately responsible for production of high affinity antibodies and immunological memory (59, 60). Within GCs, B cells undergo class-switching and evolve towards a higher affinity for antigen through a process termed affinity maturation. B cells with the highest relative affinity for the target immunogen are then provided survival and maturation signals from cognate TFH cells to differentiate into memory B cells or plasma cells. Accordingly, we investigated whether the differential antibody and inhibitor response to FVIII in B6 and S129/B6 FVIII deficient mice was due to disparities in the ability to produce a GC B cell response to FVIII. To do this, FVIII specific B cells from B6 and S129/B6 FVIII deficient mice were enriched one-week post treatment with saline or challenge with FVIII using the FVIII B cell tetramer. Bound fractions were stained with GL7 and CD38 for detection of GL7^+^ CD38^lo/-^ GC B cells as well as IgM and IgD for identification of IgM^-^ IgD^-^ CSW B cells (**Figures 3A, B**).

S129/B6 FVIII deficient mice generated an enhanced GC B cell response to FVIII compared to B6 FVIII deficient mice (**Figure 3A**). Unexpectedly, 50% of B6 FVIII deficient mice either failed to develop a detectable GC B cell response to FVIII above that of background control saline treated mice or the number of FVIII reactive GC B cells were miniscule (median: 21). Consistent with the GC response, S129/B6 FVIII deficient mice produced a considerable number of FVIII reactive CSW B cells (**Figure 3B**). However, B6 FVIII deficient mice did not form a FVIII reactive CSW B cell response above that of background control saline treated mice. The failure of B6 FVIII deficient mice to generate an adequate GC B cell response to FVIII was not due to an inherent inability of these mice to produce GC reactions, as B6 FVIII deficient mice developed a detectable GC and CSW B cell response to HEL one week following transfusion of HEL SRBCs (**Figures 3C, D**). Rather, B6 FVIII deficient mice formed a greater number of GC and CSW B cells reactive to HEL compared to S129/B6 FVIII deficient mice, further highlighting the impact of non-MHC genetic differences on the humoral immune response to a blood borne antigen. In addition, the inadequate GC B cell response to FVIII in B6 FVIII deficient mice was likely not due to differences in the capacity of FVIII specific B cells in these mice to respond to FVIII, as the IgM response to FVIII was similar between B6 and S129/B6 FVIII deficient mice (**Supplementary Figure 2**). Taken together, these data suggest that the disparate anti-FVIII IgG and inhibitor response observed in B6 and S129/B6 FVIII deficient mice was due to differences in the ability of these mice to generate a GC response to FVIII.

### CD4 T Cell Response to an Immunodominant Epitope in FVIII Does Not Differ in FVIII Deficient Mice on Distinct Genetic Backgrounds

Given that TFH cells are essential for the generation and maintenance of GCs (61) and the absence of CD4 T cells abrogates the development of inhibitors (7, 8, 11), we hypothesized that the inability of B6 FVIII deficient mice to form an adequate GC B cell response to FVIII is due to insufficient activation of a TFH cell response to FVIII. To test this, the FVIII specific CD4 T cell response in immunized B6 and S129/B6 FVIII deficient mice was evaluated using a FVIII MHC Class II tetramer (I-A^b^ : FVIII_2210-2229_) that consist of SA-PE linked to 4 identical MHC Class II molecules (I-A^b^) loaded with the peptide TASSYFTNMFATWSPSKARL, an immunodominant epitope within the C2 domain of FVIII (47). As the I-A^b^ : FVIII_2210-2229_ molecules were linked to SA-PE, anti-PE magnetic microbeads were similarly used to enrich for FVIII specific CD4 T cells from B6 and S129/B6 FVIII deficient mice one week following treatment with saline or challenge with FVIII (**Supplementary Figure 3**). Enriched CD4 T cells were identified as cells lacking non-T cell lineage markers (CD11b, CD11c and CD19) and CD8 but expressing the T cell markers CD3 and CD4. Bound fractions were also stained with CD44 to detect FVIII experienced CD4 T cells as well as Bcl6, a critical transcription factor for programming of TFH cells (62–64).

**Figure 4:**
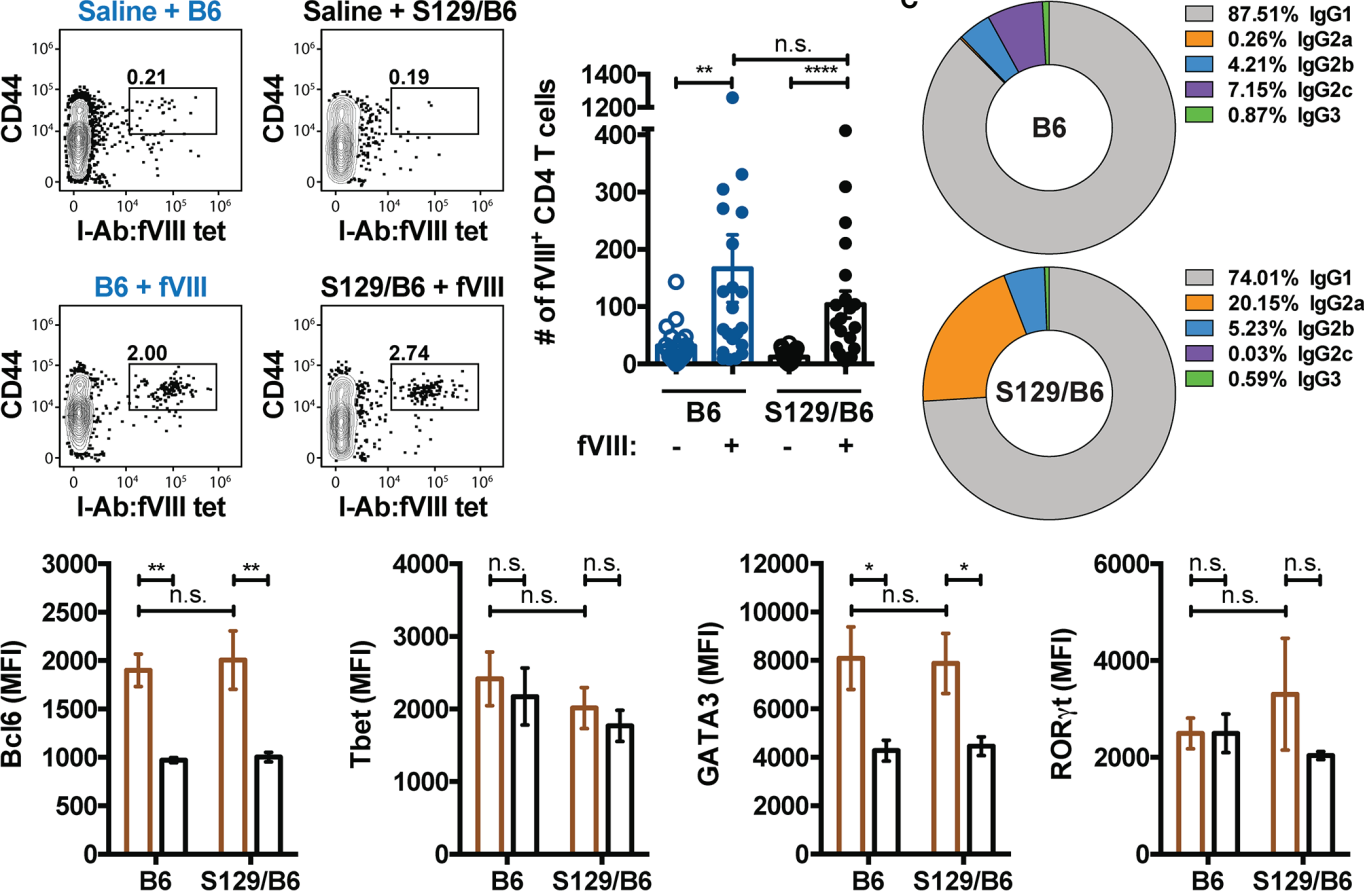
B6 and S129/B6 FVIII deficient mice develop a comparable CD4 T cell response to FVIII. **(A)** Gating strategy and graphical demonstration of the absolute counts of FVIII reactive CD4 T cells in B6 and S129/B6 FVIII deficient mice administered saline or 4 weekly infusions of 1 μg FVIII, followed by a 2 μg challenge. Splenocytes were collected 7 days post challenge, and antigen experienced FVIII specific CD4 T cells were enriched as well as quantified using the FVIII MHC Class II tetramer (I-A^b^ : FVIII_2210-2229_). **(B)** FVIII specific CD4 T cells enriched using the FVIII MHC Class II tetramer were intracellular stained for transcription factors Bcl6, Tbet, GATA3 and RORγt to assess the differentiation of the CD4 T cell response to FVIII. Mean fluorescent intensity (MFI) values of the indicated transcription factors in FVIII MHC Class II tetramer positive (brown) versus FVIII MHC Class II tetramer negative (black) CD4 T cells were used to evaluate the polarization of the CD4 T cell response. **(C)** Percentage of IgG subclasses (IgG1, IgG2a, IgG2b, IgG2c, and IgG3) in the total IgG response of B6 and S129/B6 FVIII deficient mice administered 4 weekly infusions of 1 μg FVIII, followed by a 2 μg challenge. Plasma was collected one-week post challenge and IgG subclasses were measured by ELISA. Error bars represent ± SEM. Statistics were generated using a Kruskal-Wallis with a Dunn’s multiple comparison post-test. There were 18-20 mice per group in panels **(A, C)**, and 10-11 mice per group in panel **(B)**. The results shown are the combined results from 2-4 experiments. *p < 0.05, **p < 0.01, ****p < 0.0001, and n.s. indicates not significant.

Unexpectedly, B6 and S129/B6 FVIII deficient mice developed a comparable number of activated CD4 T cells specific to an epitope in the C2 domain of FVIII (**Figure 4A**). Moreover, FVIII MHC Class II tetramer positive CD4 T cells in both strains of FVIII deficient mice demonstrated upregulated expression of Bcl6 compared to FVIII MHC Class II tetramer negative CD4 T cells (**Figure 4B**). These data suggest that the inadequate formation of a GC B cell response to FVIII in B6 FVIII deficient mice was not due to insufficient activation of a TFH cell response to an epitope in the C2 domain of FVIII. However, as mice on disparate genetic backgrounds can produce polarizing types of effector CD4 T cell responses to the exact same immunogen (65–68), we next evaluated whether B6 and S129/B6 FVIII deficient mice differ in the type of CD4 T cell response induced to FVIII. To evaluate this, samples were intracellular stained with transcription factors Tbet, GATA3 and RORγt for identification of a type 1 (T_H_1), type 2 (T_H_2) and type 17 (T_H_17) response, respectively. Consistent with previous studies (50, 69), B6 and S129/B6 FVIII deficient mice generated a dominant T_H_2 response to FVIII, as indicated by the absence of Tbet and RORγt expression but upregulation of GATA3 in FVIII MHC Class II tetramer positive CD4 T cells compared to FVIII MHC Class II tetramer negative CD4 T cells (**Figure 4B**). In support of this, examination of the IgG subclass response to FVIII demonstrated that B6 and S129/B6 FVIII deficient mice predominantly produced an IgG1 response to FVIII that is known to associate with T_H_2 responses (**Figure 4C**). Combined, these data indicate that the differential B cell response observed in B6 and S129/B6 FVIII deficient mice was likely not due to differences in the CD4 T cell response to FVIII.

## Discussion

The disparate ability to form a GC B cell response in B6 and S129/B6 FVIII deficient mice to FVIII (**Figures 1** and **3**) may in part explain the apparent propensity of patients to form distinct inhibitor signatures (12–14) as well as indicate a role of genetic factors in this process (70). For instance, the recent SIPPET (Survey of Inhibitors in Plasma-Product Exposed Toddlers) study demonstrates that the inhibitor response to plasma-derived FVIII differs between patients with low and high risk *F8* variants (2). While these results certainly implicate a role of *F8* variants in the immunogenicity of FVIII, the genetic diversity of these patients makes it difficult to determine the contributing genetic modifiers. However, as the preclinical models of hemophilia A used in the present study encode the same MHC haplotype and *F8* pathogenic variant, it is unlikely these genes contributed to the observed discordant immune response. These findings do not exclude the possibility that when present MHC or *F8* variants may also contribute to the immunogenicity of FVIII. Rather, the current data demonstrate that apart from MHC and *F8* there are yet to be identified genetic determinants capable of modulating the process by which inhibitors develop, and thereby the inhibitor signature. In particular, the ability of FVIII to induce GCs in S129/B6 FVIII deficient mice but not in those on a B6 background (**Figure 3**) suggest that these genetic factors may influence the capacity to develop a GC B cell response to FVIII. As B6 FVIII deficient mice generated a substantial population of GC B cells reactive to HEL following HEL SRBC transfusion (**Figure 3**), the failure to produce a GC response to FVIII in B6 FVIII deficient mice is not due to an inherent genetic defect in the ability to form GCs. The present results thereby demonstrate that the B cell response to FVIII in B6 FVIII deficient mice may have occurred through a non-GC dependent process, and that genetic factors outside of MHC and *F8* may play an immunological role in regulating the pathway by which humoral immunity to FVIII occurs.

Classically, the B cell response to a T cell dependent antigen like FVIII is thought to occur through a GC reaction that is often, but not always, preceded by a short phased extrafollicular response (71–75). Following antigenic encounter, B cells migrate to the interface between the T and B cell zone where B cells present antigen peptides on MHC Class II molecules to cognate CD4 T cells that have been primed by antigen presenting cells. Productive interactions lead to class-switch recombination of the BCR and clonal expansion as well as CD4 T cell differentiation into GC TFH cells. A subset of these B cells traffic to the marginal sinus bridging channels at the border of the T cell zone and red pulp to differentiate into memory B cells or plasma cells producing the 1^st^ wave of antibodies with low to modest affinity (57, 74, 76, 77). Concurrently, another fraction of B cells from the initial expansion return to the B cell follicle to work in collaboration with GC TFH cells and follicular dendritic cells to support a GC reaction, where committed GC B cells undergo somatic hypermutation and selection as well as differentiation into affinity matured, CSW memory B cells or long-lived plasma cells (71, 78, 79). Consistent with this, humoral immunity to FVIII in S129/B6 FVIII deficient mice associated with the formation of FVIII reactive GC and CSW B cells (**Figure 3**), suggesting that the B cell response in these mice may have occurred through a canonical GC pathway. However, in contrast to this classical paradigm, B6 FVIII deficient mice developed antibodies and inhibitors to FVIII in the absence of a sizeable GC response, with 50% of immunized B6 FVIII deficient mice generating a minor number of GC B cells and the other lacking a detectable GC response altogether (**Figure 3**). These results suggest that the B cell response to FVIII in B6 FVIII deficient mice may primarily occur through a pathway distinct from the GC pathway and is hypothesized to be an extrafollicular response. Corroborating these results, there is growing evidence that humoral immunity to certain immunogens can occur through an extrafollicular response and in the absence of GCs. In particular, the extrafollicular response has been reported to play a significant role in production of pathogenic antibodies in autoimmunities (80–83), formation of alloantibodies to a model red blood cell antigen HOD (45) and neutralizing antibodies to pathogens like SARS-CoV-2 (84, 85). In some of these settings, a small population of B cells expressing markers consistent with a GC phenotype have similarly been detected by flow cytometry, though these cells were reported to lack anatomical features of a GC. Taken together, these results demonstrate that inhibitors can occur through either a conventional GC or non-canonical extrafollicular response, though future studies directly testing the role of each pathway in these FVIII deficient mice is warranted. Moreover, the ability of FVIII to induce inhibitors in B6 FVIII deficient mice in the absence of an adequate GC response suggest that genetic factors may possess the ability to regulate whether the same antigen mediates a humoral immune response through a GC or extrafollicular dependent pathway.

The mechanisms responsible for directing B cells down the GC or extrafollicular pathway remain currently unknown. However, recent studies indicate that the precursor frequency of antigen specific B cells in conjunction with BCR signaling strength may regulate GC fitness (52, 53). For instance, BALB/c mice that have a lower precursor frequency of PE reactive B cells than B6 mice have been reported to generate a more robust GC B cell response due to lower avidity for PE (52). Similarly, the lower number of FVIII specific B cells in the pre-immune repertoire of S129/B6 FVIII deficient mice associated with the formation of GC B cells reactive to FVIII compared to B6 FVIII deficient mice that had a higher precursor frequency of FVIII reactive B cells but did not form a GC response (**Figures 2**, **3**). As these FVIII deficient mice encode the same *F8* pathogenic variant, it is unlikely differences in presentation of CRM during B cell selection in the bone marrow led to the decreased number of FVIII specific B cells in the pre-immune repertoire of S129/B6 FVIII deficient mice. Thus, one inference of these results is that BCR signaling strength in combination with the precursor frequency of FVIII specific B cells drives the type of B cell response induced to FVIII. However, as the number of FVIII specific B cells in the pre-immune repertoire of these mice only marginally differ, we posit that the precursor frequency of FVIII specific B cells may not be biologically relevant at least in the context of driving B cell participation in the GC response. This does not rule out the possibility that affinity or BCR signaling strength on its own may contribute to this process (86, 87). While B cells with a relatively higher initial affinity for cognate antigen are steered toward the extrafollicular pathway, those with a lower affinity for the same antigen are primarily directed to GCs. Conversely, decreasing the initial BCR affinity or antigen density reduces the extrafollicular response while having minimal impact on the ability to generate GCs. However, the theory of affinity directing the B cell response has long been controversial, with studies using NP-CGG (4-hydroxy-3-nitrophenylacetyl-chicken gamma globulin) as a model antigen suggesting that the fate of a B cell is a stochastic process that operates independent of differences in antigen recognition; B cells with a range of initial BCR affinities for NP were reported to localize in both the GC and extrafollicular foci (88–90).

Even though CD4 T cells are indispensable for B cell commitment and integration into the GC, B6 and S129/B6 FVIII deficient mice produced a comparable CD4 T cell response to FVIII. As these mice generated a similar number of activated FVIII specific CD4 T cells (**Figure 4**), the inability of B6 FVIII deficient mice to form an adequate GC B cell response to FVIII was likely not due to differences in the quantitative availability of CD4 T cells to help cognate B cells during early T-B cell interactions. Moreover, the differential B cell response does not appear to be due to differences in the effector phenotype of CD4 T cells, as FVIII specific CD4 T cells from immunized B6 and S129/B6 FVIII deficient mice expressed transcription factors Bcl6 for TFH cell differentiation and GATA3 for T_H_2 polarization (**Figure 4**). However, it is worth noting that TFH cell differentiation is a multistage process that begins with CD4 T cells upregulating Bcl6 (pre-GC TFH) following productive signals from dendritic cells (91). Bcl6 expression at this time upregulates CXCR5 and downregulates CCR7, allowing for migration of these primed pre-GC TFH cells toward the T-B cell border. Here, antigen experienced B cells can provide pre-GC TFH cells with the necessary signals to fully differentiate into TFH cells. Thus, whether Bcl6 expression in FVIII specific CD4 T cells in immunized B6 and S129/B6 FVIII deficient mice represent GC derived TFH cells or pre-GC TFH cells remains to be determined. As recent studies report a role of pre-GC TFH cells in extrafollicular responses (92, 93) and GC derived TFH cells are required to support GCs, we predict that Bcl6^+^ CD4 T cells detected in B6 FVIII deficient mice represent pre-GC TFH cells while those in S129/B6 FVIII deficient mice reflect GC derived TFH cells. Moreover, as the nature of CD4 T cell help required to promote extrafollicular responses may differ from that necessary to drive GCs, these data do not rule out the possibility that functional differences in FVIII specific pre-GC TFH cells exist and that this disparity may contribute to the ability of B cells to enter the extrafollicular or GC pathway in B6 and S129/B6 FVIII deficient mice. Nevertheless, these findings suggest that the failure to develop a GC B cell response to FVIII in B6 FVIII deficient mice is not due to insufficient activation of FVIII specific CD4 T cells or the type of effector response induced to FVIII.

As no immunological tools currently exist to examine the *in vivo* CD4 T cell response to FVIII, an MHC Class II tetramer that recognizes CD4 T cells reactive to an immunodominant epitope within FVIII (47) was used. Indeed, the use of a FVIII MHC Class II tetramer only provides insight into how a single clone reacts to FVIII exposure, and thereby may not reflect the otherwise polyclonal response (47). However, the FVIII MHC Class II tetramer allows for an *in vivo* snapshot of the ongoing immune response to FVIII. Moreover, analysis of the IgG subclass response to FVIII demonstrates that both B6 and S129/B6 FVIII deficient mice produce an IgG1 dominant response to FVIII that is indicative of a T_H_2 response and parallels the MHC Class II tetramer data demonstrating expression of the transcription factor GATA3 in FVIII MHC Class II tetramer positive CD4 T cells (**Figure 4**). Furthermore, the ability of these mice to form a T_H_2 response, inferred from both subclass and tetramer data, is consistent with clinical and preclinical studies demonstrating a similar anti-FVIII IgG subclass phenotype and the presence of T_H_2 supporting cytokines like IL4 (50, 69). Thus, while inherent limitations of the FVIII MHC Class II tetramer certainly exist and these findings do not rule out the possibility that differences in the overall polyclonal CD4 T cell response to FVIII may exist, the present data suggest that the FVIII MHC Class II tetramer can serve as a unique tool to directly track and characterize the *in vivo* CD4 T cell response to FVIII.

The clinical relevance of the current study is that the ability of FVIII to induce humoral immunity through a GC or extrafollicular pathway depending on the genetic environment not only offers unprecedented mechanistic insight into how patients may develop distinct inhibitor signatures (12–14) but also the role of genetics in this process. Moreover, these data may provide an explanation for why certain therapeutic approaches to eradicate inhibitors and/or induce immune tolerance fail in some patients with hemophilia A (15–17). Furthermore, the ability of B6 FVIII deficient mice to generate low inhibitor titers in the absence of an adequate GC B cell response may also shed light on why antibodies in patients without inhibitors have a relatively low to medium affinity for FVIII compared to those in patients with inhibitors that exhibit higher affinity (13). Indeed, the lower affinity of antibodies in patients without inhibitors may simply reflect antibodies that recognize epitopes that do not impact the procoagulant activity of FVIII. However, it is equally conceivable that these lower affinity antibodies are the sequela of a non-canonical extrafollicular B cell response. Nonetheless, while the ability to directly extrapolate our findings to the clinical setting remains to be determined, the results of the present study afford potential insight into key genetic and immune factors regulating whether humoral immunity to FVIII occurs through a GC or non-canonical extrafollicular pathway. The ability of genetic background to impact the type of immune response induced to an immunogen has certainly been well established for decades (65–68). However, the extent to which genetics influence the mechanism by which humoral immunity occurs to a therapeutic protein like FVIII in the absence of an adjuvant has remained unknown. Thus, while the identification of genetic factors regulating the pathway by which inhibitors form is outside the scope of the current study, we posit that studies using the models described herein will assist in resolving the specific determinants and mechanisms involved in shaping the *in vivo* B cell response to FVIII and other therapeutic proteins; elucidation of such factors is not ethically feasible or logistically possible to evaluate in a detailed fashion in patients.

In summary, the findings of the present study demonstrate that genetic factors outside of MHC and *F8* may be important determinants that dictate whether FVIII mediates humoral immunity through a GC or extrafollicular response. Moreover, these results suggest that in addition to a GC dependent process, inhibitors may develop through a non-canonical extrafollicular pathway. As these findings were in a murine model, testing the hypothesis in a human setting would be required before any clinical conclusions can be drawn. Nonetheless, the current data are relevant to the treatment of patients with hemophilia A and disorders requiring treatment with therapeutic proteins in that these results suggest an underappreciated immunological pathway by which humoral immunity may form. Moreover, the preclinical models utilized in the study herein hold great promise for identification of biomarkers and development of novel approaches to not only prevent humoral immunity to FVIII and other therapeutic proteins but also for autoimmune diseases as well as optimization of vaccine strategies.

## Data Availability Statement

The original contributions presented in the study are included in the article/**Supplementary Material**. Further inquiries can be directed to the corresponding author.

## Ethics Statement

This study was carried out in accordance with the recommendations of the Emory Division of Animal Resources and the Institutional Animal Care and Use Committee (IACUC). The protocol was approved by the IACUC at Emory University.

## Author Contributions

SP, SS, and SM designed the research study. SP, TL, WB, and CC carried out and analyzed experiments together with EP, JH, RJ, PZ, CJ, CD, and SM. SP and SM wrote the manuscript, which was additionally edited and commented on by the others. All authors contributed to the article and approved the submitted version.

## Funding

Research reported in this study was supported in part by the Pediatrics/Winship Flow Cytometry Core of Winship Cancer Institute of Emory University, Children’s Healthcare of Atlanta and NIH/NCI under the award P30CA138292. This study was supported by funding from the R01 (HL141335) and U54 (HL141981), as well as Hemophilia of Georgia awarded to SLM and the Judith Graham Pool Postdoctoral Award to SP.

## Conflict of Interest

SM serves on the advisory board for Genentech, Sanofi Genzyme, Takeda, Biomarin, and CSL Behring. SM consults for Pfizer and Spark, as well as receives research funding from Octapharma, Genentech. RJ is the CEO and part-owner of Biconcavity Inc, as well as owns stock in BioMarin Pharmaceutical Inc.

The remaining authors declare that the research was conducted in the absence of any commercial or financial relationships that could be construed as a potential conflict of interest.

## Publisher’s Note

All claims expressed in this article are solely those of the authors and do not necessarily represent those of their affiliated organizations, or those of the publisher, the editors and the reviewers. Any product that may be evaluated in this article, or claim that may be made by its manufacturer, is not guaranteed or endorsed by the publisher.
