# Supplementary material for: Neutralizing Antibodies Against Factor VIII Can Occur Through a Non-Germinal Center Pathway
Source: Front Immunol. 2022 May 11;13:880829. doi: 10.3389/fimmu.2022.880829 (PMC9132091; doi:10.3389/fimmu.2022.880829)
Supplement: Supplementary file 1 [file DataSheet_1.pdf]

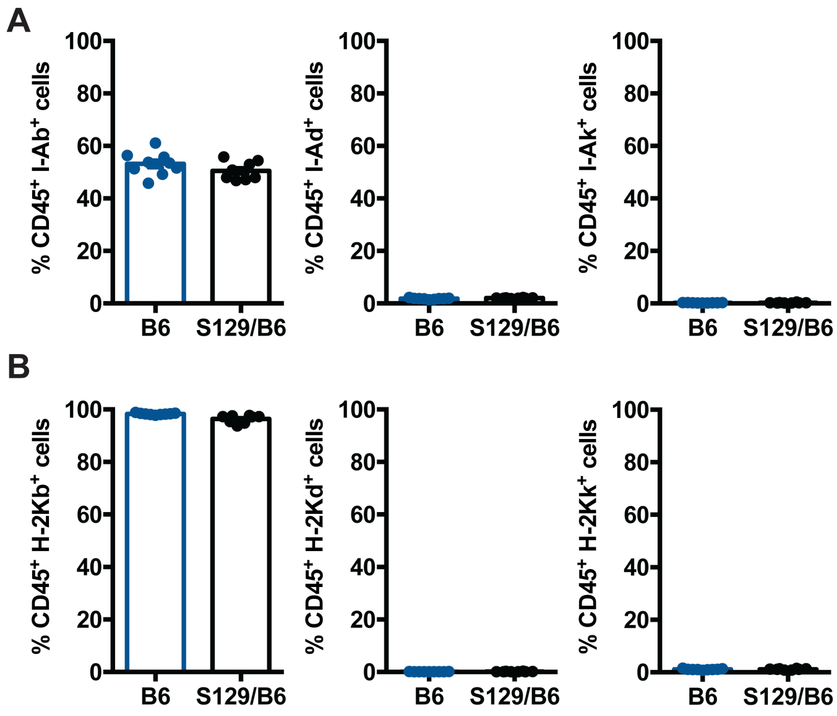

Supplemental Figure 1. MHC Class I and II haplotypes are similar in B6 and S129/B6 FVIII deficient mice. Peripheral blood leukocytes from naïve B6 and S129/B6 fVIII deficient mice were stained with the pan lymphocyte marker CD45 and antibodies that recognize distinct **(A)** MHC Class II (I-Ab, I-Ad, and I-Ak) or **(B)** MHC Class I (H-2Kb, H-2Kd, and H-2k) haplotypes. Error bars represent ± SEM.
