# Supplementary material for: Neutralizing Antibodies Against Factor VIII Can Occur Through a Non-Germinal Center Pathway
Source: Front Immunol. 2022 May 11;13:880829. doi: 10.3389/fimmu.2022.880829 (PMC9132091; doi:10.3389/fimmu.2022.880829)
Supplement: Supplementary file 2 [file DataSheet_2.pdf]

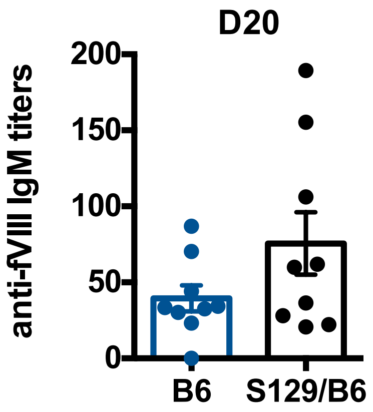

Supplemental Figure 2. IgM response to FVIII is comparable between B6 and S129/B6 FVIII deficient mice. B6 and S129/B6 FVIII deficient mice were administered 4 weekly infusions of 1 μg FVIII, followed by a 2 μg challenge. Plasma was collected one week post the 3rd exposure to FVIII (day 20 = D20) and evaluated for production of anti-FVIII IgM by ELISA. Error bars represent ± SEM. Statistics were generated using an unpaired Mann-Whitney test. Data shown are the combined results from 2 experiments.
