# Supplementary material for: Neutralizing Antibodies Against Factor VIII Can Occur Through a Non-Germinal Center Pathway
Source: Front Immunol. 2022 May 11;13:880829. doi: 10.3389/fimmu.2022.880829 (PMC9132091; doi:10.3389/fimmu.2022.880829)
Supplement: Supplementary file 3 [file DataSheet_3.pdf]

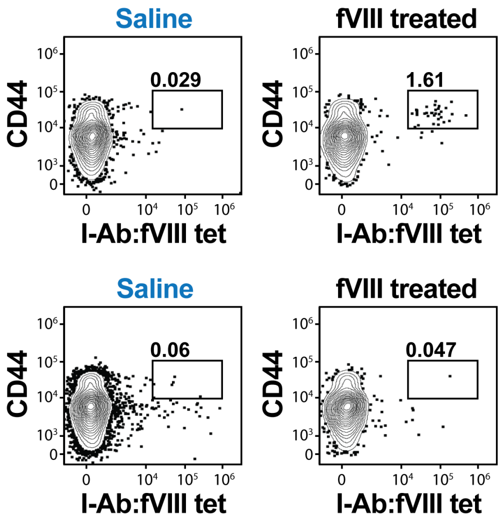

Supplemental Figure 3. The MHC Class II (I-Ab:FVIII2210-2229) tetramer identifies FVIII experienced CD4 T cells. Representative flow cytometric plots demonstrating the ability of the MHC Class II (I-Ab:FVIII2210-2229) tetramer to detect FVIII experienced CD4 T cells. Splenocytes from FVIII deficient mice immunized against FVIII or treated with saline were combined and split into 2 groups per condition. Samples were then stained with an MHC Class II (I-Ab:FVIII2210-2229) tetramer loaded with an immunodominant epitope within the C2 domain of FVIII or a non-specific CLIP tetramer, as well as CD44 to detect FVIII experienced CD4 T cells.
